# Loss of Interleukin-13-Receptor-Alpha-1 Induces Apoptosis and Promotes EMT in Pancreatic Cancer

**DOI:** 10.3390/ijms23073659

**Published:** 2022-03-26

**Authors:** Jingwei Shi, Xiao Shen, Qi Kang, Xing Yang, Maximilian Denzinger, Marko Kornmann, Benno Traub

**Affiliations:** Department of General and Visceral Surgery, Ulm Univsersity Hospital, Albert-Einstein-Allee 23, 89081 Ulm, Germany; shijingwei555@126.com (J.S.); xiao.shen@uni-ulm.de (X.S.); qi.kang@uni-ulm.de (Q.K.); xing.yang@uni-ulm.de (X.Y.); Maximilian.Denzinger@uniklinik-ulm.de (M.D.); Marko.Kornmann@uniklinik-ulm.de (M.K.)

**Keywords:** interleukin 4, interleukin 13, interleukin-13-receptor-alpha-1, cytokines, EMT, pancreatic cancer progression

## Abstract

In search of new therapies for pancreatic cancer, cytokine pathways have attracted increasing interest in recent years. Cytokines play a vital role in the crosstalk between tumour cells and the tumour microenvironment. The related inflammatory cytokines IL-4 and IL-13 can regularly be detected at increased levels in the microenvironment of pancreatic cancer. They share a receptor heterodimer consisting of IL-4Rα and IL-13Rα1. While IL-4Rα induces a more oncogenic phenotype, the role of IL-13Rα1 was yet to be determined. ShRNA-based knockdown of IL-13Rα1 was performed in Capan-1 and MIA PaCa-2. We assessed cell growth and migratory capacities under the influence of IL-13Rα1. Pathway alterations were detected by immunoblot analysis. We now have demonstrated that the loss of IL-13Rα1 induces apoptosis in pancreatic cancer cells. This was associated with an epithelial-to-mesenchymal transition. Loss of IL-13Rα1 also abolished the effects of exogenous IL-4 and IL-13 stimulation. Interestingly, in wild type cells, cytokine stimulation caused a similar increase in migratory capacities as after IL-13Rα1 knockdown. Overall, our results indicate the vital role of IL-13Rα1 in the progression of pancreatic cancer. The differential expression of IL-4Rα and IL-13Rα1 has to be taken into account when considering a cytokine-targeted therapy in pancreatic cancer.

## 1. Introduction

Pancreatic cancer (PC) remains one of the carcinomas with the worst prognosis [1]. Most patients diagnosed with pancreatic cancer show aggressive local growth combined with rapid development of distant metastases, where innovative surgical and medical treatments are urgently needed [2,3]. For promising approaches to detect and cure pancreatic cancer, it is crucial to understand the tumour development and progression, where the tumour microenvironment (TME) has received increasing attention [4,5,6]. The characteristic desmoplastic reaction of PC originates from a heterogeneous composition of the TME including mesenchymal and immune cells, as well as a dense collagen-based tumour stroma [7].

The TME components were shown to influence the malignant behaviour of PC [8,9,10]. One major impact are cytokines released by both tumour cells directly and by TME components such as cancer-associated fibroblasts (CAFs) and tumour-associated macrophages (TAMs) [11,12,13], as they contribute to aggressive cancer progression, metastasis, and suppression of tumour-directed immune surveillance mechanisms [14,15,16].

Among those cytokines, the interleukin (IL)-4/IL-13 cytokine-receptor system [17,18] has been shown promote cancer cell survival, invasion, and metastasis [19,20,21] both directly as well as via interactions with various immunoregulatory cells, such as TAMs and mast cells [22].

IL-4 and IL-13 act on pancreatic cancer cells mainly through their receptor heterodimers IL-4-receptor-alpha (IL-4Rα) and IL-13Rα1, termed type II IL-4R, via signal pathways of STAT3/6, IRS-ERK/PI3K-Akt and mTOR [23]. IL-4 can also bind to the type I receptor complex, comprising of IL-4Rα and the common gamma chain (γc) (IL-4/IL-4Rα/γc) [13], predominantly expressed on hematopoietic cells.

In our previous studies, the expression of IL-4 and IL-13 ligands, as well as IL-4Rα and IL-13Rα1 receptor chains, was shown in pancreatic cancer cell lines [24,25,26,27]. Exogenous IL-4 and IL-13 enhanced the growth of pancreatic cancer cells in a dose-dependent manner [25,26], which was inhibited by IL-4-/IL-13-neutralizing antibodies. Furthermore, overexpression of IL-13 in pancreatic cancer tissues and the high co-expression of IL-13 and IL-4Rα correlated with a higher risk of lymph node metastasis [26]. Additionally, the inhibition of IL-4Rα in Capan-1 reduced cell proliferation and migration [27].

Previous results are indicative of a contributing role of the IL-13/IL-13Rα1 axis to pancreatic cancer. However, the isolated effects of the IL-13Rα1-receptor chain on the malignant phenotype of pancreatic cancer cells and the underlying mechanisms were not studied yet.

## 2. Results

### 2.1. Expression of IL-13Rα1, IL-4Rα and γc Chains in Cultured Human Pancreatic Cancer Cells

**Figure 1:**
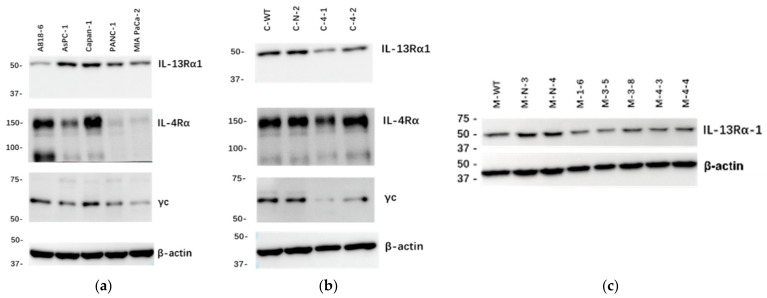
Immunoblot analysis of IL-13Rα1, IL-4Rα and γc chains. Expression of IL-13Rα1, IL-4Rα, and γc was determined in cultured pancreatic cell lines A818-6, AsPC-1, Capan-1, PANC-1 and MIA PaCa-2 (**a**), and sham-transfected clone C-N-2 and C-KD clones (**b**) by Western blot; (**c**) Expression of IL-13Rα1 chain in M-WT, sham-transfected clones and M-KD clones. Representative blot of 3 independent experiments was shown. β-actin was used as loading control. Abbreviations: C-KD: Capan-1-IL-13Rα1-knockdown; C-N: Capan-1 sham-transfected cells; M-KD: MIA PaCa-2-IL-13Rα1-knockdown; M-N: MIA PaCa-2 sham-transfected cells.

The protein levels of IL-13Rα1, IL-4Rα and γc in cultured human pancreatic cancer cell lines A818-6, AsPC-1, Capan-1, PANC-1 and MIA PaCa-2, were determined by Western blot (WB). All pancreatic cancer cell lines expressed IL-13Rα1 (47 kDa), IL-4Rα (140 kDa) and γc (64 kDa) at various levels (Figure 1a). There, Capan-1 expressed the highest level of IL-13Rα1 and was thus chosen as target cell line for IL-13Rα1 knockdown (KD). MIA PaCa-2 expressed moderate level of IL-13Rα1 but the least levels of both IL-4Rα and γc, which indicated MIA PaCa-2 also as an attractive target cell line, potentially being more dependent of IL-13Rα1.

ShRNA-based transfection targeting IL-13Rα1 was used to generate clones with reduced expression of the receptor chain. WB was performed to verify the downregulation of IL-13Rα1 with the highest efficacy in clones C-4-1 and C-4-2 (Figure 1b and Figure S1a,b). Sham-transfected clones C-N-2 and C-N-3 showed no difference in IL-13Rα1 expression compared with Capan-1 wild type (C-WT). In MIA PaCa-2, immunoblotting revealed high efficacy of IL-13Rα1-downregulation in clones M-1-6, M-3-5, M-3-8, M-4-3 and M-4-4. Sham-transfected clones M-N-3 and M-N-4 showed no difference in IL-13Rα1 expression compared with MIA PaCa-2 wild type (M-WT) and were used as control clones in further experiments (Figure 1c).

Furthermore, IL-4Rα expression showed no difference between C-WT, C-N-2, C-4-1, and C-4-2 (Figure 1b and Figure S1c). Interestingly, the expression of γc was decreased in C-KD clones after transfection (Figure 1b and Figure S1d).

### 2.2. Effects of IL-13Rα1-Downregulation on the Malignant Phenotype in Pancreatic Cancer Cells

#### 2.2.1. Effect of IL-13Rα1-Downregulation on Cell Growth

**Figure 2:**
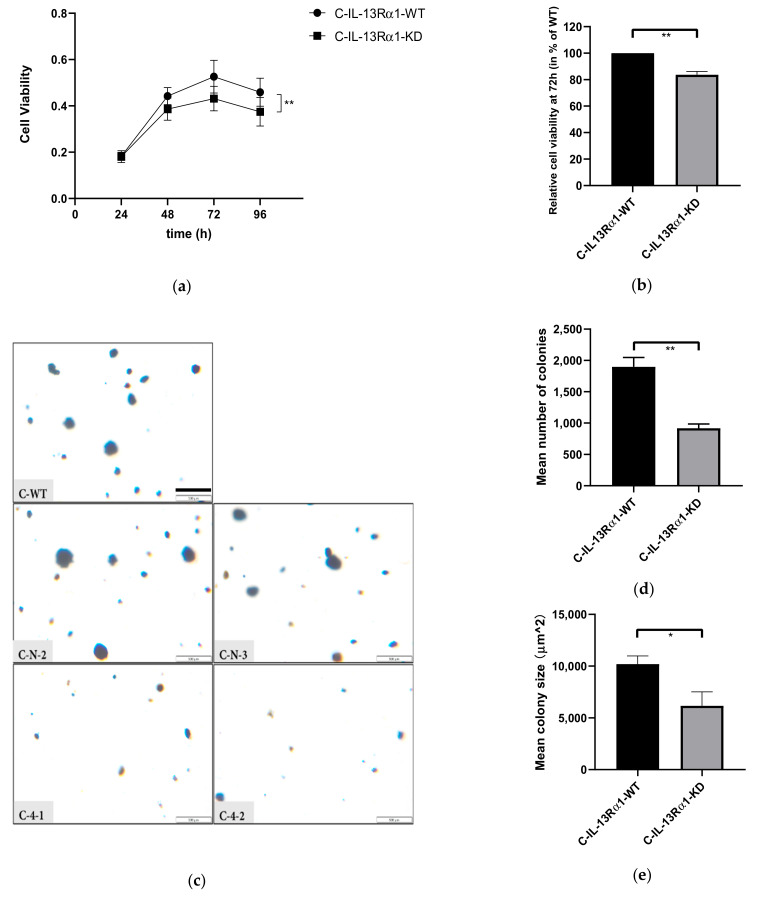
Effect of IL-13Rα1-downregulation on the basal growth of Capan-1 cells. (**a**,**b**) Anchorage-dependent growth in the MTT assay. (**a**) Cell viability at 24, 48, 72 and 96 h. The absorbance values at 570 nm detected in the MTT assay represent the cell viability and are shown as means of 3 independent experiments of quadruplicate determinations. There is an increasing difference in cell growth of Capan-1 cells with normal IL-13Rα1 expression (C-IL-13-Rα1-WT) and KD clones in a time-dependent manner; (**b**) Relative cell viability at 72 h. Data are shown as mean cell viability in % (±SEM) compared to C-IL-13-Rα1-WT and are means of 4 independent experiments of quadruplicate determinations; (**c**–**e**) Anchorage-independent growth in the colony formation assay; (**c**) Colonies formed in soft agar. Representative pictures show colonies formed by C-WT, C-N-2, C-N-3, C-4-1 and C-4-2 cells growing in soft agar after 21 days at 4x magnification. Scale bar: 500 μm. Pictures of colonies in 6-well plates were evaluated at random position in each of 9 fields per well. Colonies of C-WT and C-N were more abundant and larger in size; (**d**) Mean number of colonies lager that 50 μm^2^ (±SEM) and (**e**) mean colony size of the largest 10 colonies µm^2^ (±SEM) in one well (9.4 cm^2^) of a 6-well plate. Colony number and size were automatically calculated using ImageJ 1.52a. Data shown are means of 3 independent experiments (* *p* < 0.05, ** *p* < 0.01). Abbreviations: C-IL-13-Rα1-WT: Biological replicates with normal IL-13Rα1 expression (WT and sham transfected Neo clones), C-IL-13-Rα1-KD: Biological replicates with reduced IL-13Rα1 expression (C-4-1 and C-4-2).

The effect of IL-13Rα1-downregulation on pancreatic cancer cell proliferation was investigated by cell viability assay (MTT assay) and colony formation assay. The results indicated an increasing difference in viable cells between control groups and C-KD clones in the MTT assay over time (Figure 2a). After 72 h, significantly less viable cells were detected for clones C-4-1 and C-4-2 compared to clones C-N-2 and C-N-3 (*p* < 0.0001, Figure 2b and Figure S6a). There was no significant difference among C-WT and C-N clones. Furthermore, as shown in the soft agar assay (Figure 2c,d and Figure S6b,c), the number, as well as the size of the colonies formed by pancreatic cancer cells in soft agar after 21 days, was decreased in the IL-13Rα1-KD clones.

**Figure 3:**
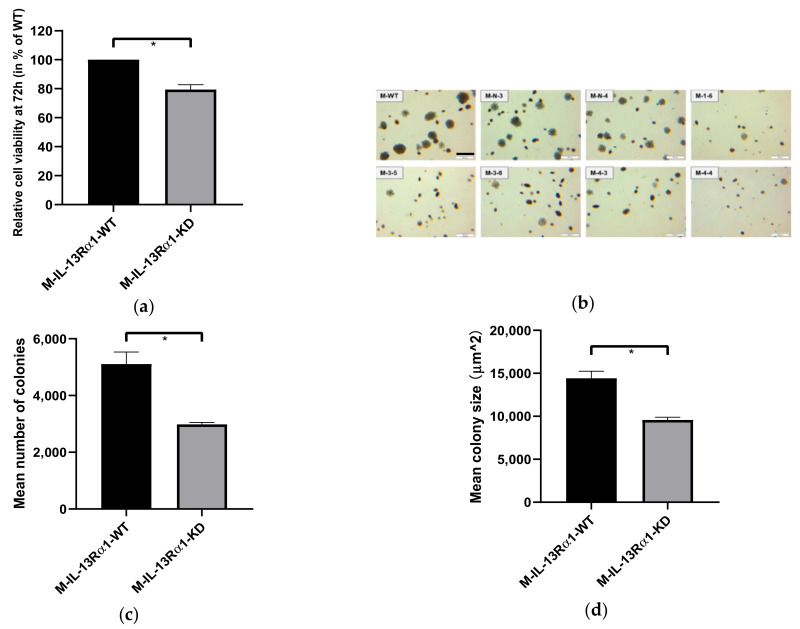
Effect of IL-13Rα1-downregulation the basal growth of M-WT, M-N and M-KD clones. (**a**) Basal anchorage-dependent growth of MIAPaCa-2 control groups and IL-13Rα1-downregulated clones in the MTT assay. Relative cell growth at 72 h is shown as mean cell viability in % (±SEM) compared to M-IL13-Rα1-WT and are means of 5 independent experiments of sextuplicate determinations; (**b**) Colonies formed in soft agar. Representative pictures show colonies formed by M-WT, M-N-3, M-N-4, M-1-6, M-3-5, M-3-8, M-4-3 and M-4-4 cells growing in soft agar after 21 days at 4x magnification. Pictures of colonies in 6-well plates were taken at random position in each of 9 fields per well. Scale bar: 500 μm. M-WT and M-N cells formed more and larger sizes of colonies; (**c**) Mean number of colonies (±SEM) and (**d**) mean colony size in µm^2^ (±SEM) in one well (9.4 cm^2^) of 6-well plate. Data shown were performed as means of 3 independent experiments. (ns *p* > 0.05, * *p* < 0.05). These findings were replicable in MIA PaCa-2 (Figure 3 and Figure S6d–f). Thus, IL-13Rα1-downregulation reduced pancreatic cancer cell survival in both anchorage-dependent and -independent assays. Abbreviations: M-IL-13-Rα1-WT: Biological replicates with normal IL-13Rα1 expression (WT and sham transfected Neo clones), M-IL-13-Rα1-KD: Biological replicates with reduced IL-13Rα1 expression (M-1-5, M-3-5, M-3-8, M-4-3, M-4-4).

These findings were replicable in MIA PaCa-2 (Figure 3 and Figure S6d–f). Thus, IL-13Rα1-downregulation reduced pancreatic cancer cell survival in both anchorage-dependent and -independent assays.

#### 2.2.2. Effect of IL-13Rα1-Downregulation on the Cell Cycle

**Figure 4:**
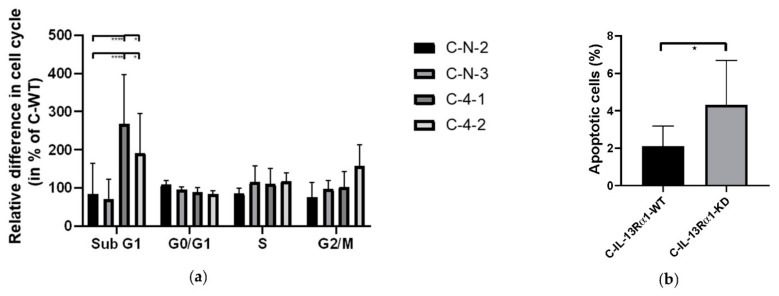
Cell cycle and apoptosis analysis. (**a**) Cell cycle analysis. Data acquired by Flow Cytometer and analysed by Flowjo are shown as relative difference of cell cycle fraction of C-N and C-KD clones in % (±SEM) compared to C-WT and are means of five independent experiments. The percentage of Sub G1 represents the fraction of cells in apoptosis. (**b**) Staining of Annexin V in Capan-1 cells indicates higher percentages of apoptotic cells after IL-13Rα1 knockdown. Results are shown as means of 3 independent experiments. (* *p* < 0.05, **** *p* < 0.0001).

Cell cycle analysis (Figure 4a) showed no significant difference in cell cycle progression through G0/G1, S, and G2/M phase. However, C-4-1 and C-4-2 cells showed high fractions of cells in the sub-G1 phase. We can therefore conclude that the results seen before are not due to reduced proliferation but rather that the loss of IL-13Rα1 induces apoptosis of Capan-1 cells. We confirmed an increase in apoptotic cells by Annexin V staining (Figure 4b). No consistent alterations were shown in alternative cell death pathways (S7)

#### 2.2.3. Effect of IL-13Rα1-Downregulation on Cell Mobility and Migration

**Figure 5:**
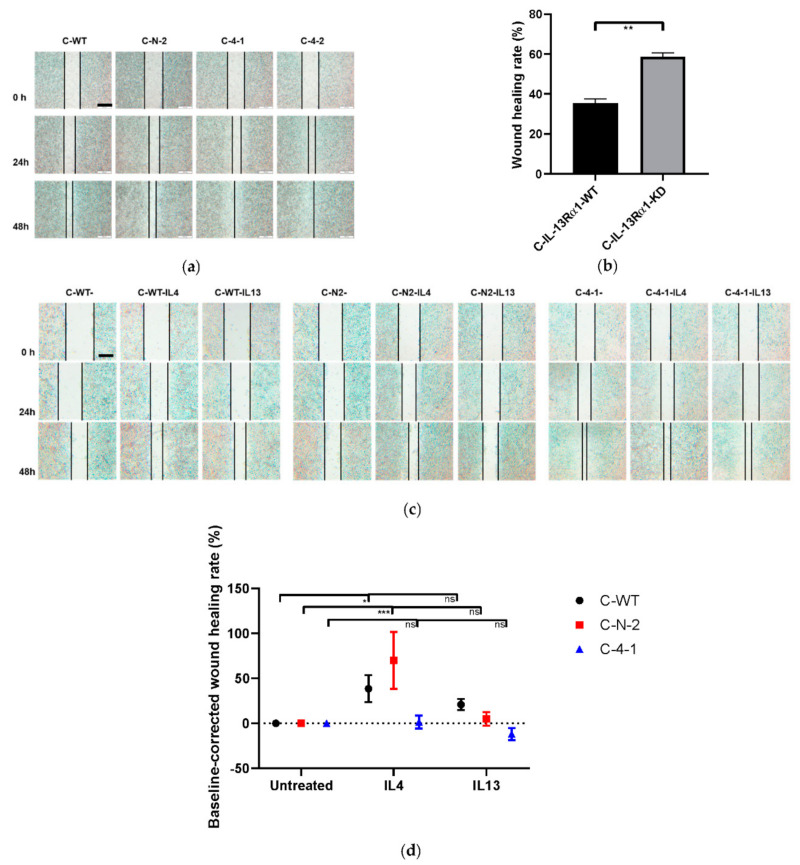
Cell mobility in the wound healing assay. (**a**,**b**) Effect of IL-13Rα1-downregulation on cell mobility. (**a**) Wound healing of C-WT, C-N-2, C-4-1 and C-4-2. Representative pictures shown were recorded at 0, 24 and 48 h after scratch. C-4-1 and C-4-2 managed wound closure at 48 h; (**b**) Wound healing rate. Data are shown as means ± SEM of (A-B)/A × 100% (A is the wound gap at 0 h and B is the wound gap at 24 h after scratch) of C-IL-13-Rα1-WT and C-IL-13-Rα1-KD cells and are means of 3 independent experiments of quadruplicate determinations; (**c**,**d**) Effect of exogenous IL-4 and IL-13 on wound healing. Cells were cultured in full medium with or without IL-4 (1nM) and IL-13 (1nM), respectively after the scratch. Representative pictures shown were recorded at 0, 24 and 48 h (**c**); (**d**) Baseline-corrected wound healing rates after 24 h IL-4 and IL-13 treatment. The baseline represents the wound closure rate for each cell line untreated. Data are displayed as 100% × (A-baseline)/baseline (A represents the wound healing rate of respective group) and were obtained from 3 independent experiments. Scale bar: 500 μm. (ns *p* > 0.05, * *p* < 0.05, ** *p* < 0.01, *** *p* < 0.001).

Migratory capacities were tested in the scratch assay first. As shown, wound healing rates of C-4-1 and C-4-2 were significantly higher than C-WT and C-N-2 (Figure 5a). Wounds of IL-13Rα1-KD clones, unlike the control groups, were closed at 48h, which indicates that IL-13Rα1-downregulation enhances the mobility of pancreatic cancer cells (Figure 5b and Figure S8a). Wound healing rates of control groups and C-KD clones were further investigated after the treatment with recombinant IL-4 and IL-13. As shown, exogenous IL-4, but not IL-13 increased the wound healing rates of C-WT and C-N clones (Figure 5c,d). Wound closure of IL-13Rα1-KD clone C-4-1 was not affected by exogeneous IL-4 or IL-13.

**Figure 6:**
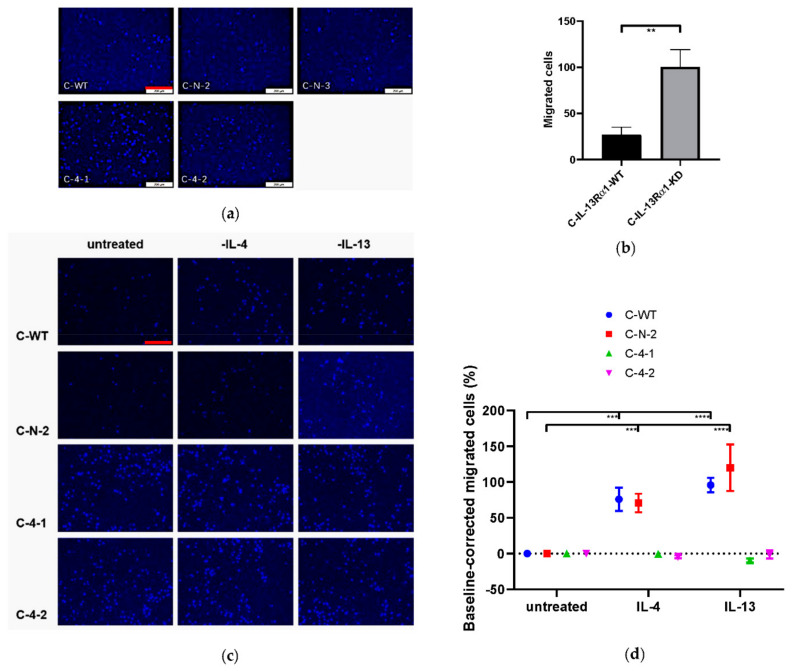
Directed migration in the modified Boyden-Chamber-Assay. (**a**,**b**) Effect of IL-13Rα1-downregulation on cell migration. (**a**) Representative pictures of C-WT, C-N-2, C-N-3, C-4-1 and C-4-2 show DAPI-labelled migrated cells within 24 h. 4 pictures were taken from each membrane at random positions; (**b**) Migrated cells per high power field (HPF). Data are shown as mean number of migrated cells of C-IL-13-Rα1-WT and C-IL-13-Rα1-KD clones within 24 h (±SEM) and are means of 6 independent experiments. IL-13Rα1-downregulation remarkably increased the directed migration of Capan-1 cells; (**c**, **d**) Effect of IL-4 (1nM) and IL-13 (1nM) treatment on directed migration. (**c**) Representative pictures in dependency of exogenous IL-4 and IL-13 treatment; (**d**) Number of migrated cells after IL-4 and IL-13 treatment per HPF. Results are shown as baseline-corrected mean numbers (±SEM) of migrated cells within 24 h after treatment with IL-4 and IL-13. The baseline represents the cell migration for each cell line untreated. It is displayed as 100% × (A—baseline)/baseline (A represents the mean number of cells in respective group). Data are means of 4 independent experiments. Both IL-4 and IL-13 significantly enhanced the directed migration of C-WT and C-N-2 compared to the untreated controls. Scale bar: 200 μm. (** *p* < 0.01, *** *p* < 0.001, **** *p* < 0.0001).

Furthermore, the directed migration of C-WT, C-N and C-KD clones was accessed in the Boyden chamber assay. Consistently, the migratory capacity of the IL-13Rα1-KD clones were increased (Figure 6a,b). Migration was increased 4.5-fold in C-4-1 and 2.5-fold in C-4-2 clones, respectively, compared to control cells (*p* < 0.0001) (Figure S8b).

Similar to above, the influence of exogenous IL-4 and IL-13-treatment was determined. Again, IL-4, but also IL-13 treatment, significantly increased the directed migration of cells with normal IL-13Rα1 expression but was without effect on C-KD clones (Figure 6c).

#### 2.2.4. Effect of IL-13Rα1-Downregulation on Epithelial-to-Mesenchymal Transition (EMT)

**Figure 7:**
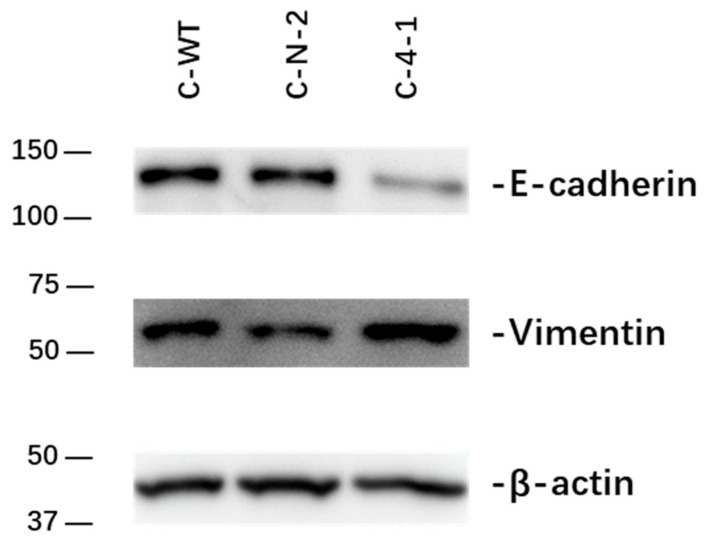
Immunoblot analysis of E-cadherin and vimentin in C-WT, C-N and C-KD clones. WB was performed to determine the expression of E-cadherin and vimentin in C-WT, C-N-2 and C-4-1 cells**.** β-actin was used as loading control. Downregulation of IL-13Rα1 leaded to reduced E-cadherin expression and upregulated the expression of vimentin.

With altered migratory capacities, the switch from an epithelial to a mesenchymal phenotype is a common finding. Morphologically, no changes were observed in Giemsa staining (Figure S2). On a cellular level, E-cadherin and vimentin are critically involved markers in EMT [28]. The expression of E-cadherin and vimentin was compared in C-WT, C-N-2 and C-4-1 (Figure 7**)**. Interestingly, we found lower expression of E-cadherin and higher expression of vimentin in C-4-1 compared to C-WT and C-N-2, suggesting a more mesenchymal phenotype.

### 2.3. Effect of IL-13Rα1-Downregulation on IL-4 and IL-13 Signalling

**Figure 8:**
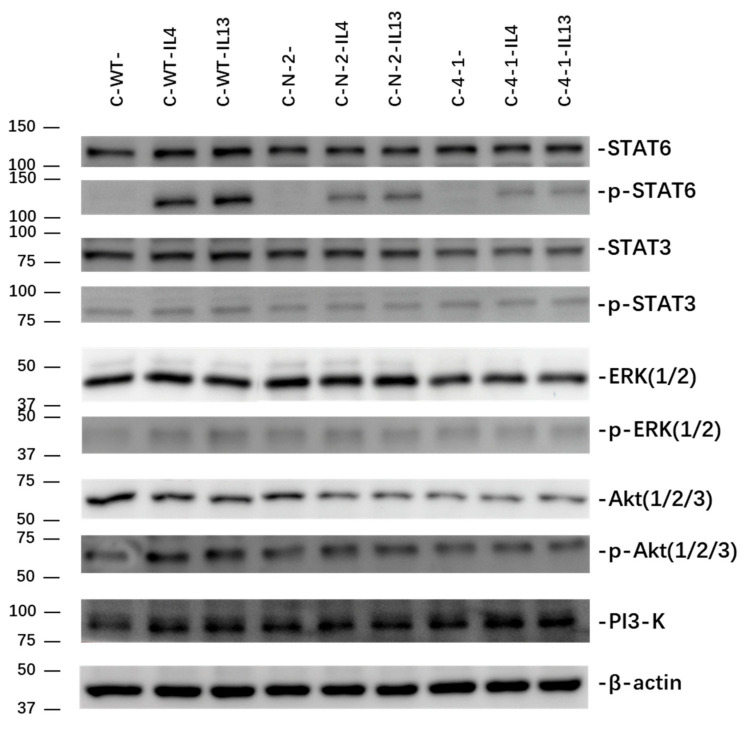
Basal and phosphorylated expression of STAT3, STAT6, ERK1/2 and Akt, and activation of PI3K in C-WT, C-N and C-KD clones. WB was performed to determine the expression of relevant pathway components in C-WT, C-N-2 and C-4-1. The phosphorylation of kinases and transcription factors was detected in cells treated with recombinant IL-4 (0.4 nM for 30 min) and IL-13 (1 nM for 30 min) in comparison to untreated cells (-). β-actin was used as loading control. Abbreviations: Akt: protein kinase B; ERK: extracellular signal-regulated kinase; IRS: insulin receptor substrate; PI3K: phosphoinositide 3-kinase; STAT: signal transducer and activator of transcription.

Changes in the downstream signalling of the IL-4 and IL-13 axis in pancreatic cancer cells were investigated in Capan-1(Figure 8). Baseline expression of relevant pathway components of IL-4 and IL-13 signalling (STAT3, STAT6, ERK1/2, Akt, and PI3K) were examined in Capan-1 WT cells as well as control clone C-N-2 and the IL-13Rα1 knockdown clone C-4-1. The respective pathway activation was determined by protein phosphorylation after treatment with IL-4 (0.4 nM for 30 min) and IL-13 (1 nM for 30 min).

Control transfected C-N-2 showed comparable or even increased baseline expression of the analysed pathway components with C-WT cells. In the loading-control corrected expression of STAT6, STAT3, ERK and PI3K were comparable to control cells. However, C-4-1 showed a markedly reduced baseline expression of Akt (Table 1, Figure 8).

Expectedly, exogenous cytokine stimulation resulted in strong pathway activation downstream of IL-4Rα and IL-13Rα1. The phosphorylation of STAT6 is the most prominent with very low baseline expression and strong activation after stimulation. Similarly, but not to the same extent, all other pathways showed increased phosphorylation.

The phosphorylation of STAT6 was markedly reduced after knockdown of IL-13Rα1, indicating the disrupted signalling through the Type II IL-4-receptor. With reduced baseline expression, phosphorylation levels of Akt were also reduced. STAT3, ERK and PI3K showed comparable activation levels after cytokine stimulation irrespective of IL-13Rα1 expression (Table 2, Figure 8)

## 3. Discussion

IL-4 and IL-13 were initially identified as pleiotropic T helper 2 cytokines with overlapping, but distinct functions in multiple immune and inflammatory events [29,30,31]. Now, increasing evidence indicates salient activities of IL-4, IL-13 and their specific receptor complex IL-4Rα/IL-13Rα1 in carcinomas including pancreatic cancer [19,23]. The expression of IL-4 and IL-13, as well as IL-4Rα and IL-13Rα1 receptor chains, was shown in several cultured pancreatic cancer cell lines by us and by other research groups [24,26,27,32,33]. Exogenous IL-4 and IL-13 enhanced the growth of pancreatic cancer cells [25,26], while IL-4-/IL-13-neutralizing antibodies counteracted this effect [18].

In human samples, IL-13 was not expressed in the physiological pancreatic compartments (ductal, acinar, islets) but showed immunoreactivity in 43% of PDAC specimen and the high co-expression of IL-13 and IL-4Rα was associated with an increased risk for lymph node metastasis [26]. By analysing sequencing data from the International Cancer Genome Consortium (ICGC) from the study groups PACA-CA and -AU (Pancreatic Cancer Canadian and Australian), we found mutations in 44 of 659 patients. However, only in two cases, clinically significant single base substitutions were found, resulting in one missense mutation and one gained STOP-codon [34]. On the contrary, in differential gene expression datasets of human PDAC, IL-13Rα1 IL-4Rα and the common γc chain was consistently upregulated compared to normal controls. Dey and colleagues furthermore showed that upregulation of IL-13Rα1, IL-4Rα and γc was dependent on KRAS expression and loss of KRAS was associated with reduced receptor chain expression [35]. While the tumour promoting role of IL-4Rα on pancreatic cancer cells in vitro and in vivo has been shown before, the role and mechanism of action of IL-13Rα in pancreatic cancer is yet unclear.

In order to further investigate the role of IL-13Rα1, we screened the protein expression of IL-13Rα1 and IL-4Rα in 5 PC cells lines. Interestingly, exogenous IL-13 significantly enhanced the growth of AsPC-1 and Capan-1 cells, with high IL-13Rα1 expression, while PANC-1 and MIA PaCa-2, with low IL-13Rα1 levels, were less responsive [24,26], indicating a positive impact of the IL-13-IL-13Rα1 axis on cell survival and growth.

This is contrary to IL-4, where IL-4-enhanced cell proliferation was independent of IL-4Rα expression, although IL-4-coupled toxin was more efficient in cell lines with high IL-4Rα expression [24]. Thus, not only the distinct expression levels of respective receptor chains, but also the ratio between IL-13Rα1 and IL-4Rα expression may be crucial for the effect of IL-4 and IL-13 on the cancer cell phenotype. This is of special interest as in both our previous [27] and current results, the expression of the unmodified receptor chain of the heterodimer complex IL-4Rα/IL-13Rα1 was unaltered by the downregulation of its partner. However, we were the first to show that the alternative receptor chain, the IL-2 common γ-chain was downregulated in parallel to IL-13Rα1 knockdown (KD).

IL-2 γc expression is gained in pancreatic cancer progression and ranks among the highest upregulate genes during pancreatic cancer progression [36,37]. The clinical implication of this upregulation is, however, not yet determined, as knockout of γc only moderately reduced tumour burden in vivo [35,37]. The reduced expression of γc after IL-13Rα1 knockdown in our study may be associated with a reduced oncogenic potential. However, its clinical significance warrants further studies.

In line with previous results for IL-4Rα, our data showed an inhibitory effect of IL-13Rα1-downregulation on cell viability/growth in two different pancreatic cancer cell lines. This was due to an increase in apoptotic cells, while cell cycle progression was unimpaired. Previously, our group showed that IL-13-induced growth acceleration of PC cells was associated with an increased S-phase cell fraction and reduced percentage of cells in G0/G1 [26]. Combined with our current finding with increased apoptosis after IL-13Rα1 KD, the vital role of IL-13Rα1 for PC cell survival is stressed.

IL-4 and IL-13 are believed to carry out abundant functions in tumour cells through several pro-oncogenic pathways involving signal factors such as STAT3 [25,27], STAT6 [38], PI3K/Akt [39], and ERK1/2 [39,40]. In our present study, we can confirm the involvement of STAT3, STAT6, PI3K, Akt and ERK1/2 in the response to exogenous IL-4 and IL-13. Furthermore, we demonstrated that downregulation of IL-13Rα1 in Capan-1 cells leads to a decrease in baseline expression of Akt. Furthermore, responsiveness towards ligand binding is reduced, displayed as reduced phosphorylation of mainly STAT6, Akt. As those are key mediators in regulating cell survival and growth [41,42,43], we propose that the reduced cell survival through enhanced apoptosis mechanistically is due to the suppression of STAT6 and Akt. This is in line with previous results demonstrating the activation of the Type II IL-4 receptor through IL-4 and IL-13 stimulation with phosphorylation of downstream JAK1 and STAT6 [35].

Thus, one could argue that the reduced apoptosis resistance after IL-13Rα1 KD may render the cells less cancerous. However, besides sustained proliferation and evasion of cell death, the ability to invade tissues and form metastasis is considered one of the “hallmarks on cancer” [44].

Wound healing and migration were significantly increased after IL-13Rα KD. This is paralleled by an increase in EMT markers with increased expression of Vimentin and reduced E-cadherin expression, although cellular morphology was unaltered.

Convincing evidence suggests that EMT is involved in promoting invasion and metastasis in pancreatic cancer [28]. In other visceral malignancies, IL-13 stimulation was associated with increased EMT [45,46]. Similarly, in our results, the exogenous stimulation resulted in increased cell migration. This was especially obvious in the difference between the non-directed migration in the wound healing assay and the directed migration in the Boyden chamber assay. IL-13 stimulation consistently increased migration in the Boyden chamber assay in both WT and control cells, while this effect was far less pronounced in the wound healing assay.

However, we are the first to report that the loss of IL-13Rα1 also promotes an EMT phenotype. This is a highly interesting new finding as the results of exogenous cytokine stimulation needs to be re-evaluated. Possibly, the promoted EMT phenotype through IL-4/-13 stimulation is due to cellular signalling mainly through the IL-4Rα receptor and the relative loss of IL-13Rα1 further promotes this phenotype. However, the precise mechanisms underlaying these observations require further experiments.

Up to date, IL-13-focussed treatments were studied in clinical trials, which utilized the Pseudomonas Exotoxin coupled IL-13. These trials, such as the PRECISE Trial (randomized controlled Phase III clinical trial), were conducted in Glioblastoma multiforme patients and achieved prolonged time to progression in treated patients [47]. In pancreatic cancer, IL-13 or IL-4 is not studied in clinical trials currently. However, the RECAP Trial (NCT01423604) utilizes ruxolitinib, a JAK1/JAK2-Inhibitor, targeting the downstream pathways of IL-13 and IL-4. In metastatic PDAC, ruxolitinib was able to prolong overall and progression free survival [48].

Overall, we can conclude that IL-13Rα1 is vital for cell survival and apoptosis resistance. However, its loss induces an EMT phenotype and consistently promotes cell migration. The more detailed knowledge in IL-4/-13 signalling we have received from this study helps in designing more promising clinical studies, as the multiple functions and cross-play of ligands and receptor chains need to be taken into account.

## 4. Materials and Methods

### 4.1. Cell Lines and Cell Culture

Human pancreatic cancer cell lines A818-6, AsPC-1, Capan-1 and were cultured in RPMI (Roswell Park Memorial Institute medium). MIA PaCa-2 and PANC-1 were cultured in DMEM (Dulbecco’s Modified Eagle’s Medium). All media were supplemented with 10% foetal calf serum (FCS), 1% Penicillin (10,000 U/mL)/Streptomycin (10,000 μg/mL). The maintenance media for transfected clones containing a neomycin resistance gene were supplemented with 550 and 1100 µg/mL geneticin (G418) sulphate, respectively. Cells were cultured in 100 mm cell culture dishes and maintained in monolayer culture at 37 °C in humidified air with 5% CO_2_. All cells were tested as mycoplasma-free.

### 4.2. Immunoblotting

Western blotting was performed as previously described [27]. Cultured cells at around 80% confluence were washed twice with ice-cold DPBS (Dulbecco’s Phosphate Buffered Saline) and were incubated with lysis buffer for 30 min on ice. Protein concentration was measured using the Pierce^®^ BCA Protein Assay kit (Thermo Fisher Scientific, Waltham, MA, USA). Rabbit anti-IL-13Rα1 antibody (ab79277, 1:500, Abcam, Berlin, Germany), mouse anti-IL-4Rα antibody (sc-28361, 1:100), anti-STAT6 antibody (sc-271213, 1:100), anti-Akt1/2/3 (sc-81434, 1:200), anti-p-ERK (sc-7383, 1:200), anti-PI3K (sc-1637,1:200), rabbit anti-ERK2 (sc-154, 1:200) and anti-p-Akt1/2/3(sc-7985-R, 1:200) from Santa Cruz Biotechnology (Dallas, Texas, USA), goat anti-human common γ chain (AF284, 0.1 μg/mL, R&D Systems, Minneapolis, MN, USA), rabbit anti-STAT3 antibody (#4904, 1:2000), anti-phospho-STAT3 antibody (#9131, 1:1000), anti-phospho-STAT6 (#9361, 1:1000), anti-Vimentin (#5741, 1:1000) and anti-E-Cadherin (#3195, 1:1000) from Cell Signalling Technology (Frankfurt am Main, Hesse, Germany) were used as primary antibodies. To ensure equal loading, β-actin (A5441, 1:5000, Sigma-Aldrich, Taufkirchen, Bavaria, Germany) was used as the internal control. Images were acquired by the imaging system (FUSION FX, Vilber Lourmat Deutschland GmbH, Weinheim, Baden-Württemberg, Germany) and analysed by ImageJ 1.52a (National Institutes of Health, Bethesda, MD, USA).

### 4.3. Transfection

Stable transfection was performed using Capan-1 and MIA PaCa-2 cells, 4 different plasmids, each containing one shRNA construct directed against human IL-13Rα1, 1 negative control plasmid (SureSilencing shRNA Plasmid for Human IL-13Rα1, QIAGEN, Hilden, North Rhine-Westphalia, Germany), and the Effectene Transfection Reagent Kit (QIAGEN, Hilden, North Rhine-Westphalia, Germany) and using the conditions described by the supplier. Each plasmid contains a neomycin resistance gene. After transfection, cells were cultured with the appropriate selection medium (RPMI for Capan-1 and DMEM for MIA PaCa-2 supplemented with 10% FCS, penicillin G (100 U/mL), streptomycin (100 µg/mL) and additional G418 (Capan-1: 1100 μg/mL, MIAPaCa-2: 2200 μg/mL)), until single cell colonies formed. Single cell clones were isolated and checked for IL-13Rα1-KD separately.

### 4.4. Cell Growth Assay

The basal anchorage-dependent growth of cultured cells was determined by the MTT colorimetric assay as described before [27]. Briefly, 10,000 cells/well were seeded and viable cells were detected after incubation with the MTT reagent at the absorbance of 570 nm after 24, 48, 72, and 96 h.

Colony formation assay was performed to assess the basal anchorage-independent growth of cancer cells. For the base layer, 2 mL of 0.9% agar solution was gently added into each well of a prewarmed 6-well plate. When the base agar solution was solidified, 4000 vital cells were gently resuspended in 0.35% agar solution and added onto the base layer. After the top agar solution solidified, plates were maintained at 37 °C in 5% CO_2_ atmosphere. After 21 days, 9 photos were taken per well, as shown in Figure S4. Afterwards, the mean number of colonies and mean colony size were measured by ImageJ 1.52a.

### 4.5. Cell Migration Assay

Cell movement was studied in the scratch assay. Confluent cells in a monolayer were scratched to make equidistant wounds by yellow tips, as shown in Figure S5. Distances of the wounds were recorded in quadruplicate by taking pictures at defined positions. Gap distances were quantitatively evaluated by ImageJ 1.52a. The wound healing rate was determined as (A − B)/A × 100%, where A was the primary wound width and B was the wound width after 24 or 48 h.

The modified Boyden Chamber assay was performed to investigate cell migration as mentioned before [27]. Then, 5 × 10^4^ cells suspended in 100 μL of medium containing 1% FCS were seeded into each insert, which was placed in the 24-well plate, as shown in Figure S6. Non-migratory cells were scraped off with wet cotton swabs after 24 h, while migratory cells on the underside of the membrane were rinsed by dH_2_O, fixed with 4% paraformaldehyde and stained with DAPI for 5 min. Afterwards, fluorescence photographs were taken at 6 random positions at 10x magnification. Migratory cells were counted using ImageJ 1.52a.

### 4.6. Giemsa Staining Assay

Exponentially growing cells in 100 mm dishes were rinsed by 10 mL of DPBS, fixed in 5 mL of methanol for 15 min, and then stained in Giemsa staining solution (Giemsa Stain, Sigma-Aldrich, Taufkirchen, Bavaria, Germany, diluted with dH2O in the ratio of 1:20) for 15 min. Cell morphology was observed and recorded by taking photos under an inverted light microscope.

### 4.7. Cell Cycle and Apoptosis Analysis

Cell cycle analysis was performed using Propidium Iodide staining (Sigma-Aldrich, Taufkirchen, Bavaria, Germany) and flow cytometry analysis. Data were acquired using MACSQuant^®^ X Flow Cytometer (Miltenyi Biotec, Bergisch Gladbach, North Rhine-Westphalia, Germany) and analysed by FlowJo_v10.6.1 (FlowJo LLC, Ashland, OR, USA).

Annexin V-FITC Kit (Miltenyi Biotec, Bergisch Gladbach, North Rhine-Westphalia, Germany) was used to detect apoptotic cells. Experiments were performed according to the protocol supplied by the manufacturer. Apoptotic cells are stained positively for Annexin V-FITC but are negative for staining with PI.

### 4.8. Statistics

Statistical analysis was performed using GraphPad Prism 8.0.1 (GraphPad Software, San Diego, California, USA). Paired *t* test, ratio paired *t* test, Tukey’s multiple comparisons test and uncorrected Fisher’s LSD were used for evaluating group differences. *p* values <0.05 were taken as level of significance. *p* values are shown as follows: ns *p* > 0.05, * *p* < 0.05, ** *p* < 0.01, *** *p* < 0.001 and **** *p* < 0.0001.

## 5. Conclusions

Overall, IL-13Rα1 plays a critical and diverse role in the survival and migration of cultured pancreatic cancer cells. The findings of this study may help to better understand the different functions and mechanisms involving IL-13Rα1 in pancreatic cancer progression. As cytokines such as IL-4 and IL-13 play a vital role in the interaction of tumour cells and components of the TME, their understanding is crucial in order to design better therapies for pancreatic cancer.

## Figures and Tables

**Table 1:** Baseline expression of IL-13 signalling components in percent of WT expression. Expression was determined with ImageJ 1.52a and grey-scale values were corrected by loading control.

	STAT6	STAT3	ERK	Akt	PI3K
C-WT	100	100	100	100	100
C-N-2	117.6	110.6	151.8	83.3	114.5
C-4-1	119.6	83.3	97.7	41.2	105.1

**Table 2:** Pathway activation after exogenous cytokine stimulation. Protein phosphorylation was determined with ImageJ 1.52a and grey-scale values were corrected by loading control.

	p-STAT6	p-STAT3	p-ERK	p-Akt	PI3K
C-WT-IL4	1479.3	196.9	182.8	251.2	129.4
C-WT-IL13	1769.0	225.3	209.1	275.6	133.4
C-N-2-IL4	778.7	166.2	202.3	215.6	104.4
C-N-2-IL13	837.4	148.9	162.7	211.7	126.5
C-4-1-IL4	435.9	141.2	169.9	168.4	131.9
C-4-1-IL13	348.9	114.4	180.9	140.3	145.4

## Data Availability

Not applicable.

## Supplementary Materials

The following are available online at https://www.mdpi.com/article/10.3390/ijms23073659/s1.

## Abbreviations

Akt	Protein kinase B
C-KD	Capan-1-IL-13Rα1-knockdown clone
C-N	Capan-1 sham-transfected cells
C-WT	Capan-1 wild type
DMEM	Dulbecco’s Modified Eagle’s Medium
DPBS	Dulbecco’s Phosphate Buffered Saline
EMT	Epithelial–mesenchymal transition
ERK	Extracellular signal-regulated kinase
FCS	Fetal calf serum
γc	common gamma chain
GSDMD	Gasdermin D
HPF	High power field
IL	Interleukin
IL-4Rα	IL-4-receptor alpha
IL-13Rα	IL-13-receptor alpha
IRS	Insulin receptor substrate
KD	knockdown
kDa	kilodalton
MAPK	Mitogen-activated protein kinase
MMPs	Matrix metalloproteinases
M-KD	MIA PaCa-2-IL-13Rα1-knockdown clone
M-N	MIA PaCa-2 sham-transfected cells
M-WT	MIA PaCa-2 wild type
mTOR	The mechanistic target of rapamycin
p-MLKL	phospho-mixed lineage kinase domain-like protein
PCR	Polymerase chain reaction
PC	Pancreatic cancer
PI3K	phosphoinositide 3-kinase
qPCR	real-time quantitative polymerase chain reaction
RNA	Ribonucleic acid
RPMI	Roswell Park Memorial Institute medium
SDS-PAGE	Sodium dodecyl sulfate polyacrylamide gel electrophoresis
ShRNA	Short haipin ribonucleic acid
STAT	Signal transducer and activator of transcription
TAMs	Tumor-associated macrophages
TME	Tumor microenvironment
WB	Western blot

## References

[1] Sung H., Ferlay J., Siegel R.L., Laversanne M., Soerjomataram I., Jemal A., Bray F. (2021). Global cancer statistics 2020: GLOBOCAN estimates of incidence and mortality worldwide for 36 cancers in 185 countries. CA Cancer J. Clin..

[2] Neoptolemos J.P., Kleeff J., Michl P., Costello E., Greenhalf W., Palmer D.H. (2018). Therapeutic developments in pancreatic cancer: Current and future perspectives. Nat. Rev. Gastroenterol. Hepatol..

[3] Traub B., Link K.-H., Kornmann M. (2021). Curing Pancreatic Cancer. Seminars in Cancer Biology.

[4] Lei X., Lei Y., Li J.-K., Du W.-X., Li R.-G., Yang J., Li J., Li F., Tan H.-B. (2020). Immune cells within the tumor microenvironment: Biological functions and roles in cancer immunotherapy. Cancer Lett..

[5] Parayath N., Padmakumar S., Nair S.V., Menon D., Amiji M.M. (2020). Strategies for targeting cancer immunotherapy through modulation of the tumor microenvironment. Regen. Eng. Transl. Med..

[6] Thakkar S., Sharma D., Kalia K., Tekade R.K. (2020). Tumor microenvironment targeted nanotherapeutics for cancer therapy and diagnosis: A review. Acta Biomater..

[7] Huber M., Brehm C.U., Gress T.M., Buchholz M., Alashkar Alhamwe B., von Strandmann E.P., Slater E.P., Bartsch J.W., Bauer C., Lauth M. (2020). The immune microenvironment in pancreatic cancer. Int. J. Mol. Sci..

[8] Miyabayashi K., Baker L.A., Deschênes A., Traub B., Caligiuri G., Plenker D., Alagesan B., Belleau P., Li S., Kendall J. (2020). Intraductal transplantation models of human pancreatic ductal adenocarcinoma reveal progressive transition of molecular subtypes. Cancer Discov..

[9] Wang M., Zhao J., Zhang L., Wei F., Lian Y., Wu Y., Gong Z., Zhang S., Zhou J., Cao K. (2017). Role of tumor microenvironment in tumorigenesis. J. Cancer.

[10] Whiteside T. (2008). The tumor microenvironment and its role in promoting tumor growth. Oncogene.

[11] Biffi G., Oni T.E., Spielman B., Hao Y., Elyada E., Park Y., Preall J., Tuveson D.A. (2019). IL1-induced JAK/STAT signaling is antagonized by TGFβ to shape CAF heterogeneity in pancreatic ductal adenocarcinoma. Cancer Discov..

[12] Moraga I., Richter D., Wilmes S., Winkelmann H., Jude K., Thomas C., Suhoski M.M., Engleman E.G., Piehler J., Garcia K.C. (2015). Instructive roles for agonist binding parameters in determining the functional bandwidth of cytokine receptor signaling. Science signaling.

[13] Leonard W.J., Lin J.-X., O’Shea J.J. (2019). The γc family of cytokines: Basic biology to therapeutic ramifications. Immunity.

[14] Autenshlyus A., Davletova K., Studenikina A., Mikhaylova E., Varaksin N., Zhurakovsky I., Proskura A., Sidorov S., Lyakhovich V. (2019). Cytokine production by blood immune cells, tumor and its microenvironment, characteristic of extracellular matrix in patients with invasive ductal carcinoma of no special type. Biomeditsinskaya Khimiya.

[15] Ben-Baruch A. (2002). Host microenvironment in breast cancer development: Inflammatory cells, cytokines and chemokines in breast cancer progression: Reciprocal tumor–microenvironment interactions. Breast Cancer Res..

[16] Kawaguchi K., Sakurai M., Yamamoto Y., Suzuki E., Tsuda M., Kataoka T.R., Hirata M., Nishie M., Nojiri T., Kumazoe M. (2019). Alteration of specific cytokine expression patterns in patients with breast cancer. Sci. Rep..

[17] Braddock M., Hanania N.A., Sharafkhaneh A., Colice G., Carlsson M. (2018). Potential risks related to modulating interleukin-13 and interleukin-4 signalling: A systematic review. Drug Saf..

[18] Ito S.-E., Shirota H., Kasahara Y., Saijo K., Ishioka C. (2017). IL-4 blockade alters the tumor microenvironment and augments the response to cancer immunotherapy in a mouse model. Cancer Immunol. Immunother..

[19] Hallett M.A., Venmar K.T., Fingleton B. (2012). Cytokine stimulation of epithelial cancer cells: The similar and divergent functions of IL-4 and IL-13. Cancer Res..

[20] Kwaśniak K., Czarnik-Kwaśniak J., Maziarz A., Aebisher D., Zielińska K., Karczmarek-Borowska B., Tabarkiewicz J. (2019). Scientific reports concerning the impact of interleukin 4, interleukin 10 and transforming growth factor β on cancer cells. Cent. -Eur. J. Immunol..

[21] Seyfizadeh N., Seyfizadeh N., Gharibi T., Babaloo Z. (2015). Interleukin-13 as an important cytokine: A review on its roles in some human diseases. Acta Microbiol. Immunol. Hung..

[22] Lin X., Wang S., Sun M., Zhang C., Wei C., Yang C., Dou R., Liu Q., Xiong B. (2019). miR-195-5p/NOTCH2-mediated EMT modulates IL-4 secretion in colorectal cancer to affect M2-like TAM polarization. J. Hematol. Oncol..

[23] Shi J., Song X., Traub B., Luxenhofer M., Kornmann M. (2021). Involvement of IL-4, IL-13 and Their Receptors in Pancreatic Cancer. Int. J. Mol. Sci..

[24] Kornmann M., Kleeff J., Debinski W., Korc M. (1999). Pancreatic cancer cells express interleukin-13 and-4 receptors, and their growth is inhibited by Pseudomonas exotoxin coupled to interleukin-13 and-4. Anticancer. Res..

[25] Prokopchuk O., Liu Y., Henne-Bruns D., Kornmann M. (2005). Interleukin-4 enhances proliferation of human pancreatic cancer cells: Evidence for autocrine and paracrine actions. Br. J. Cancer.

[26] Formentini A., Prokopchuk O., Sträter J., Kleeff J., Grochola L.F., Leder G., Henne-Bruns D., Korc M., Kornmann M. (2009). Interleukin-13 exerts autocrine growth-promoting effects on human pancreatic cancer, and its expression correlates with a propensity for lymph node metastases. Int. J. Colorectal Dis..

[27] Traub B., Sun L., Ma Y., Xu P., Lemke J., Paschke S., Henne-Bruns D., Knippschild U., Kornmann M. (2017). Endogenously expressed IL-4Rα promotes the malignant phenotype of human pancreatic cancer in vitro and in vivo. Int. J. Mol. Sci..

[28] Wang W., Chen H., Gao W., Wang S., Wu K., Lu C., Luo X., Li L., Yu C. (2020). Girdin interaction with vimentin induces EMT and promotes the growth and metastasis of pancreatic ductal adenocarcinoma. Oncol. Rep..

[29] Chomarat P., Banchereau J. (1998). Interleukin-4 and interleukin-13: Their similarities and discrepancies. Int. Rev. Immunol..

[30] Gandhi N.A., Pirozzi G., Graham N.M. (2017). Commonality of the IL-4/IL-13 pathway in atopic diseases. Expert Rev. Clin. Immunol..

[31] Gour N., Wills-Karp M. (2015). IL-4 and IL-13 signaling in allergic airway disease. Cytokine.

[32] Kawakami K., Kawakami M., Husain S.R., Puri R.K. (2002). Targeting interleukin-4 receptors for effective pancreatic cancer therapy. Cancer Res..

[33] Wu Y., Konaté M.M., Lu J., Makhlouf H., Chuaqui R., Antony S., Meitzler J.L., Difilippantonio M.J., Liu H., Juhasz A. (2019). IL-4 and IL-17A cooperatively promote hydrogen peroxide production, oxidative DNA damage, and upregulation of dual oxidase 2 in human colon and pancreatic cancer cells. J. Immunol..

[34] https://dcc.icgc.org/q?q=data%20obtained%20on%20February%2028th%202022.

[35] Dey P., Li J., Zhang J., Chaurasiya S., Strom A., Wang H., Liao W.T., Cavallaro F., Denz P., Bernard V. (2020). Oncogenic KRAS-Driven Metabolic Reprogramming in Pancreatic Cancer Cells Utilizes Cytokines from the Tumor Microenvironment. Cancer Discov..

[36] Rajamani D., Bhasin M.K. (2016). Identification of key regulators of pancreatic cancer progression through multidimensional systems-level analysis. Genome Med..

[37] Ayars M., O’Sullivan E., Macgregor-Das A., Shindo K., Kim H., Borges M., Yu J., Hruban R.H., Goggins M. (2017). IL2RG, identified as overexpressed by RNA-seq profiling of pancreatic intraepithelial neoplasia, mediates pancreatic cancer growth. Oncotarget.

[38] Fu C., Jiang L., Hao S., Liu Z., Ding S., Zhang W., Yang X., Li S. (2019). Activation of the IL-4/STAT6 signaling pathway promotes lung cancer progression by increasing M2 myeloid cells. Front. Immunol..

[39] Kim S.D., Baik J.S., Lee J.-H., Mun S.-W., Yi J.M., Park M.-T. (2020). The malignancy of liver cancer cells is increased by IL-4/ERK/AKT signaling axis activity triggered by irradiated endothelial cells. J. Radiat. Res..

[40] Nelms K., Keegan A.D., Zamorano J., Ryan J.J., Paul W.E. (1999). The IL-4 receptor: Signaling mechanisms and biologic functions. Annu. Rev. Immunol..

[41] Timme S., Ihde S., Fichter C., Waehle V., Bogatyreva L., Atanasov K., Kohler I., Schöpflin A., Geddert H., Faller G. (2014). STAT3 expression, activity and functional consequences of STAT3 inhibition in esophageal squamous cell carcinomas and Barrett’s adenocarcinomas. Oncogene.

[42] Datta S.R., Brunet A., Greenberg M.E. (1999). Cellular survival: A play in three Akts. Genes Dev..

[43] Nicholson K.M., Anderson N.G. (2002). The protein kinase B/Akt signalling pathway in human malignancy. Cell. Signal..

[44] Hanahan D., Weinberg R.A. (2011). Hallmarks of cancer: The next generation. Cell.

[45] Cao H., Zhang J., Liu H., Wan L., Zhang H., Huang Q., Xu E., Lai M. (2016). IL-13/STAT6 signaling plays a critical role in the epithelial-mesenchymal transition of colorectal cancer cells. Oncotarget.

[46] Gann P.H., Deaton R.J., McMahon N., Collins M.H., Dellon E.S., Hirano I., Hua S.Y., Rodriguez C., Harris S. (2020). An anti–IL-13 antibody reverses epithelial-mesenchymal transition biomarkers in eosinophilic esophagitis: Phase 2 trial results. J. Allergy Clin. Immunol..

[47] Kunwar S., Chang S., Westphal M., Vogelbaum M., Sampson J., Barnett G., Shaffrey M., Ram Z., Piepmeier J., Prados M. (2010). Phase III randomized trial of CED of IL13-PE38QQR vs. Gliadel wafers for recurrent glioblastoma. Neuro Oncol..

[48] Hurwitz H., Uppal N., Wagner S.A., Bendell J., Thaddeus B.J., Wade S., Nemunaitis J., Stella P., Pipas J.M., Wainberg Z. (2014). Results from a Phase 2 Study of Ruxolitinib or Placebo with Capecitabine as Second-Line Therapy in Patients with Metastatic Pancreatic Cancer: The Recap Trial. Ann. Oncol..

