# Supplementary material for: Loss of Interleukin-13-Receptor-Alpha-1 Induces Apoptosis and Promotes EMT in Pancreatic Cancer
Source: Int J Mol Sci. 2022 Mar 26;23(7):3659. doi: 10.3390/ijms23073659 (PMC8998778; doi:10.3390/ijms23073659)
Supplement: Supplementary file 1 [file ijms-23-03659-s001.zip › ijms-1604609-supplementary.pdf]

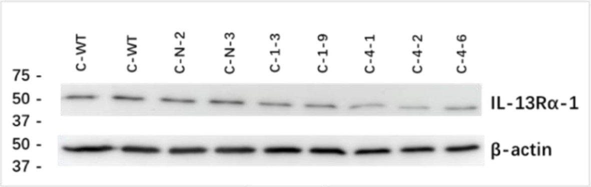

(a)

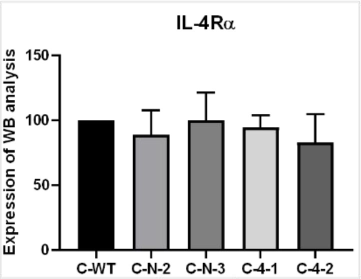

(c)

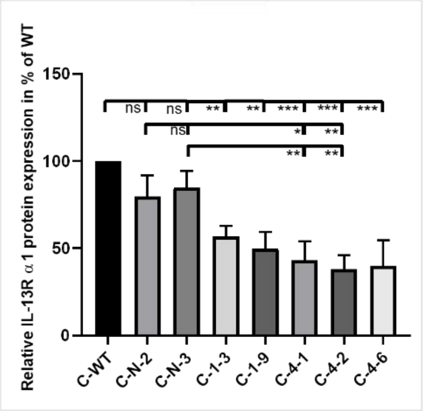

(b)

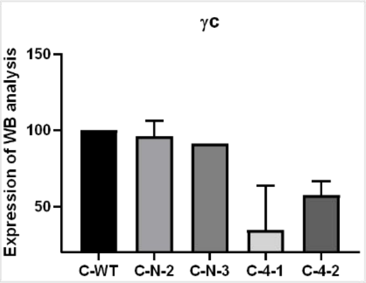

(d)

Figure S1. Expression of IL-13Rα1, IL-4Rα and γc in C-WT, C-N and C-KD clones. (**a**) Immunoblot analysis of IL-13Rα1 expressed in cell lysates. β-actin was used as loading control. One representative of 3 blots is shown; (**b**) Relative levels of IL-13Rα1 protein expressed in C-WT, C-N and C-KD clones. Results are shown as IL-13Rα1/β-actin in % (±SD) compared to C-WT and are means of 3 separate experiments (ns $p > 0.05$, * $p < 0.05$, ** $p < 0.01$, *** $p<0.001$); Quantitative expression of IL-4Rα (**c**) and γc (**d**) was abtained by analyzing 3 and 2 times of immunoblotings respectively, while GAPDH was used as internal control.

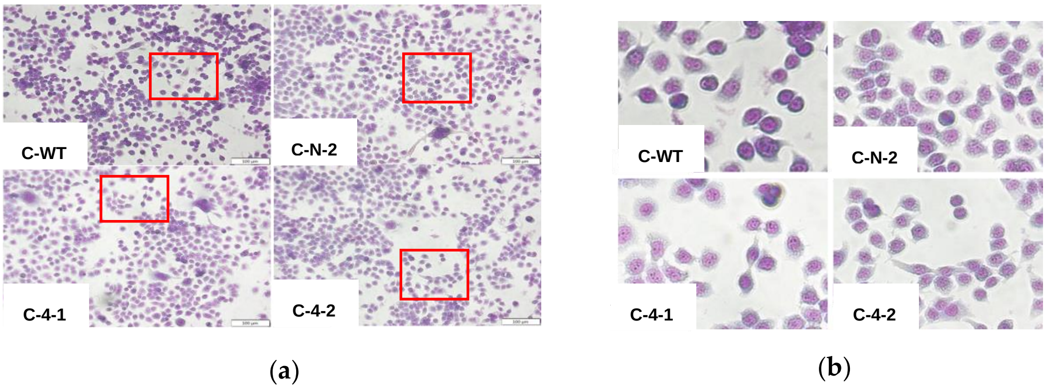

Figure S2. C-WT, C-N and C-KD cells stained by Giemsa staining solution. (**a**) Giemsa-stained C-WT, C-N-2, C-4-1 and C-4-2 clones. Photos were taken under reverse light microscope at 20x magnification. Scale bar: 100 μm. There is no significant difference in cell morphology among 4 groups; (**b**) Representative stained cells shown at 20x2x magnification. Pictures shown are amplified areas marked by red box in (a).

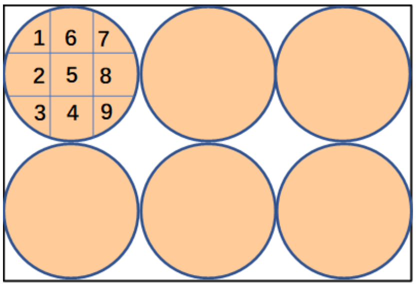

Figure S3. Positions to take photos of the 6-well plate. Photos were taken at the positions shown in a well of the 6-well plate, from 1 to 9, by moving the plate under the microscope.

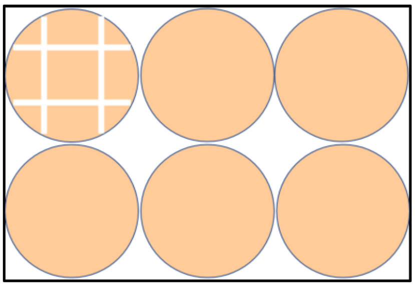

Figure S4. Wounds scratched in the 6-well plate. Primary wounds (white lines) with same gap distance were scratched on the bottom of wells attached by single-layer cells in the 6-well plate using sterile 200 μl yellow tips.

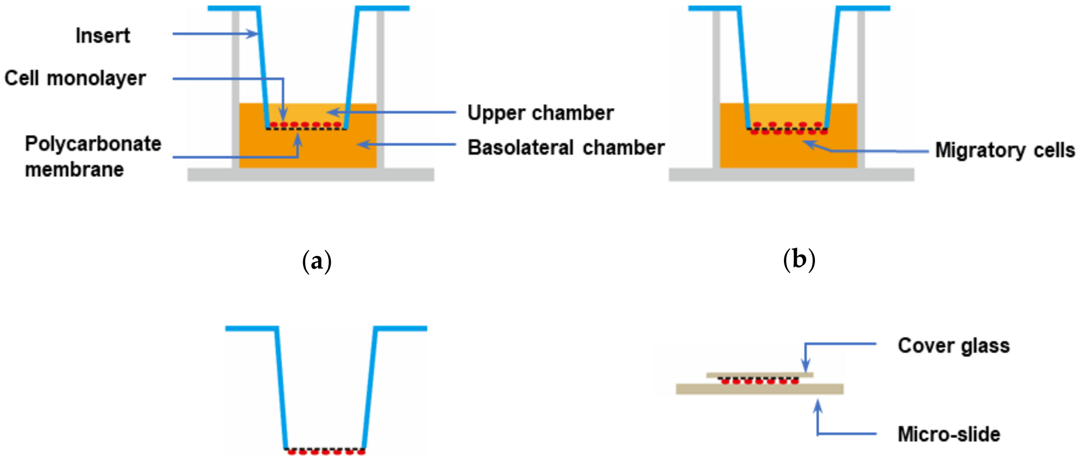

(**a**) (**b**) (**c**) (**d**)

Figure S5. Modified Boyden Chamber assay. (**a**) 5 x 10^4 cells were seed into an insert containing 100 µl of medium containing 1% FCS, while 600 µl of medium containing 10% FCS was added into the basolateral chamber; (**b**) Cells were allowed to migrate from the upper chamber with low serum to the basolateral chamber with high serum within 24 h; (**c**) Non-migratory cells on the upper side of the membrane were scraped off with wet cotton swabs, while migratory cells on the underside of the membrane were rinsed by dH_2O, fixed with 4% paraformaldehyde and stained with DAPI; (**d**) Polycarbonate membranes were cut from inserts and placed on microscope slides. 6 photos were taken at random under reverse fluorescence microscope. **Abbreviations:** FCS: fetal calf serum; DAPI: 4′,6-diamidino-2-phenylindole.

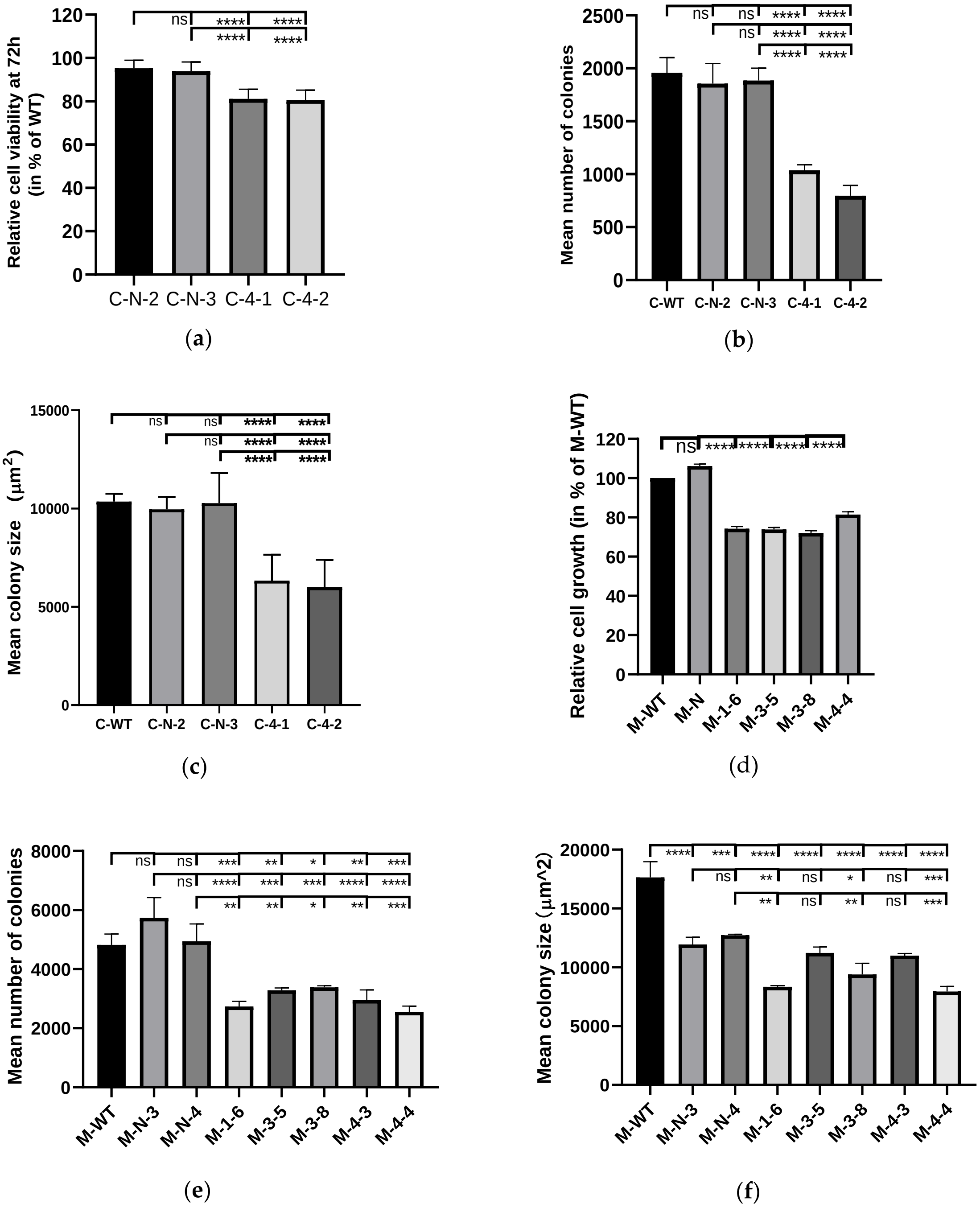

Figure S6. Effect of IL-13Rα1-downregulation on the basal growth of pancreatic cancer cells. (**a**, **d**) Anchorage-dependent growth in the MTT assay. Relative cell viability of Capan-1 cells (**a**) and MIA PaCa-2 cells (**d**) at 72 h was shown. Data are shown as mean cell viability in % (±SEM) compared to WT and are means of 4 and 3 independent experiments of quadruplicate determinations respectively. (**b-c, e-f)** Anchorage-independent growth in the colony formation assay. (**b**) Mean number of Capan-1 colonies lager that 50 μm² (±SEM) and (**c**) mean colony size of the largest 10 colonies μm² (±SEM) in one well (9.4 cm²) of a 6-well plate. Colony number and size were automatically calculated using ImageJ 1.52a. Data shown are means of 3 independent experiments. (**e**) Mean number of MIA PaCa-2 colonies (±SEM) and (**f**) mean colony size in μm² (±SEM) in one well (9.4 cm²) of 6-well plate. Data shown as means of 3 independent experiments. (ns p>0.05, * p<0.05, ** p<0.01, **** p<0.0001).

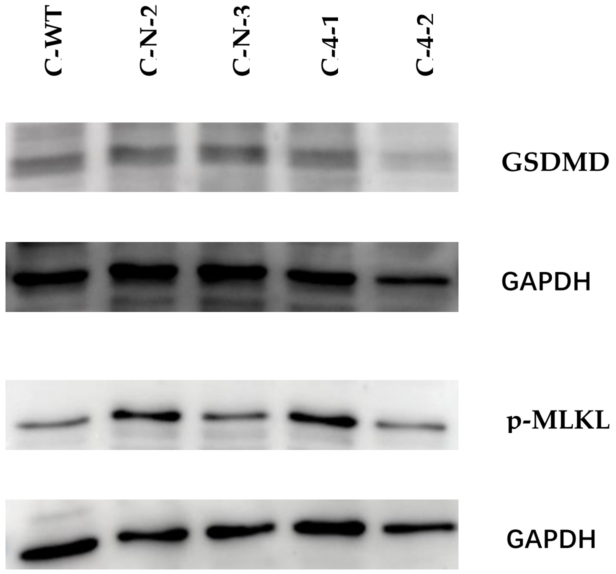

Figure S7. Immunoblot analysis of GSDMD and p-MLKL. Expression of GSDMD and p-MLKL was determined in the cell lysates of Capan-1 wild type, sham-transfected clones and Capan-1-IL-13Rα1-knockdown clones. The increase in GSDMD (Gasdermin D) and p-MLKL (phospho-mixed lineage kinase domain-like protein) indicate the induction of pyroptosis and necroptosis respectively, which was not shown in the present project.

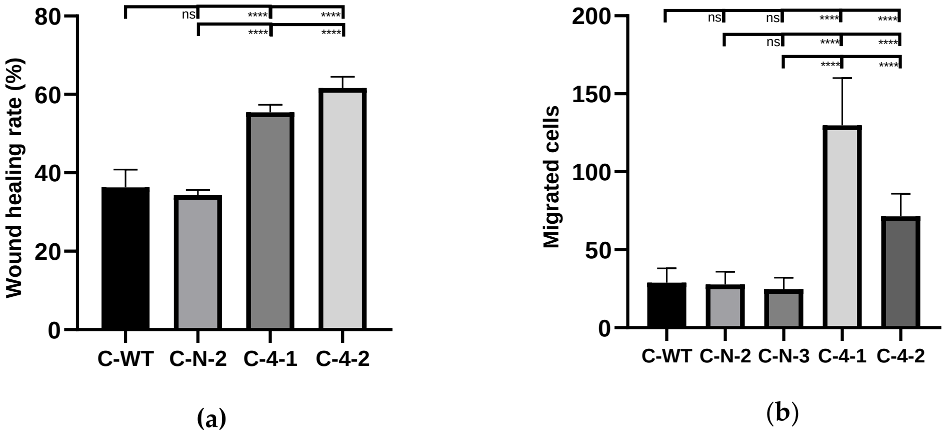

Figure S8. Effect of IL-13Rα1-downregulation on cell mobility and migration of pancreatic cancer cell clones. (**a**) Cell mobility in the wound healing assay. Wound healing rate represents the cell mobility and data are shown as means ± SEM of (A-B)/A*100% (A is the wound gap at 0 h and B is the wound gap at 24 h after scratch) and are means of 3 independent experiments of quadruplicate determinations. (**b**) Directed migration in the modified Boyden-Chamber-Assay. Results are migrated cells per high power field (HPF). Data are shown as mean number of migrated cells within 24 h (±SEM) and are means of 6 independent experiments. (ns $p>0.05$, **** $p<0.0001$).
